# Estimating the Magnitude and Direction of Altered Arbovirus Transmission Due to Viral Phenotype

**DOI:** 10.1371/journal.pone.0016298

**Published:** 2011-01-27

**Authors:** Rebecca C. Christofferson, Christopher N. Mores

**Affiliations:** Department of Pathobiological Sciences, School of Veterinary Medicine, Louisiana State University, Baton Rouge, Louisiana, United States of America; University of Texas Medical Branch, United States of America

## Abstract

Vectorial capacity is a measure of the transmission potential of a vector borne pathogen within a susceptible population. Vector competence, a component of the vectorial capacity equation, is the ability of an arthropod to transmit an infectious agent following exposure to that agent. Comparisons of arbovirus strain-specific vector competence estimates have been used to support observed or hypothesized differences in transmission capability. Typically, such comparisons are made at a single time point during the extrinsic incubation period, the time in days it takes for the virus to replicate and disseminate to the salivary glands. However, vectorial capacity includes crucial parameters needed to effectively evaluate transmission capability, though often this is based on the discrete vector competence values. Utilization of the rate of change of vector competence over a range of days gives a more accurate measurement of the transmission potential. Accordingly, we investigated the rate of change in vector competence of dengue virus in *Aedes aegypti* mosquitoes and the resulting vectorial capacity curves. The areas under the curves represent the effective vector competence and the cumulative transmission potentials of arboviruses within a population of mosquitoes. We used the calculated area under the curve for each virus strain and the corresponding variance estimates to test for differences in cumulative transmission potentials between strains of dengue virus based on our dynamic model. To further characterize differences between dengue strains, we devised a displacement index interpreted as the capability of a newly introduced strain to displace the established, dominant circulating strain. The displacement index can be used to better understand the transmission dynamics in systems where multiple strains/serotypes circulate or even multiple arbovirus species. The use of a rate of a rate of change based model of vectorial capacity and the informative calculations of the displacement index will lead to better measurements of the differences in transmission potential of arboviruses.

## Introduction

The transmission potential of a vector borne disease has been used to predict risks of outbreaks, evaluate vector control strategies, and to compare strains of a pathogen [1], [2], [3]. An accurate measure of this potential is critical, and often estimated by vectorial capacity (VC) [4]. Vectorial capacity was originally devised by MacDonald in 1957 for malariologists, and represents “the number [of infections] that a specific mosquito population can distribute per case per day [4], [5], [6], [7].” The calculation of vectorial capacity is given by:
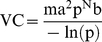
(1)where **a** is the man biting rate and **m** is the mosquito density; these parameters are measures of contact between the vector and vertebrate hosts [7]. The probability of daily survival **p** is a measure of the mortality rate of the vector [7]. The extrinsic incubation period (EIP) **N** is the time, in days, it takes for a pathogen to infect the mosquito and disseminate to the salivary glands where it can be transmitted [7]. The original formula of vectorial capacity has been modified to include a transmission capability parameter, vector competence **b** [8], [9], [10], [11], [12]. These last two parameters, EIP and b, capture intrinsic viral characteristics and have been used to evaluate differences in pathogen strains [13], [14], [15], [16]. Other researchers have recognized the importance of understanding the parameters in vectorial capacity as well as characterizing the interaction of important parameters, EIP, survival rate and vector competence, as a means for evaluating infection risks [1], [17].

Vector competence **b** is the ability of an arthropod to transmit an infectious agent following exposure to that agent [12]. Several vector traits have been studied with regard to vector competence differences, such as mosquito species, mosquito strain within species and mosquito size [18], [19], [20], [21]. Vector competence for arboviruses particularly is impacted by extrinsic factors such as temperature differences during incubation, titer of virus offered during exposure, and larval competition [22], [23], [24], [25], [26]. Estimates of vector competence can also be indicative of differences in vector susceptibility to arbovirus strains. Indeed, several studies have shown that vector competence of dengue virus varies within a single serotype [14], [15]. Vector competence is estimated as the proportion of mosquitoes with a disseminated infection to the total number of exposed mosquitoes and can therefore be thought of as the dissemination rates within a vector population.

Comparisons of arbovirus strain-specific vector competence estimates have been used to support observed or hypothesized differences in transmission capability [2], [27], [28], [29], [30]. Typically, such comparisons are made at a single (optimal) time point during the extrinsic incubation period, and less commonly two or more time points might be used [2], [13], [30], [31], [32]. Using the appropriate extrinsic incubation period (EIP) is crucial when calculating vectorial capacity [1], [2]. However, the continuous interaction between EIP and vector competence has not been evaluated in terms of vectorial capacity estimates. Further, the importance of the mosquito survival rate is also critical given as it sets time constraints on the EIP. The importance of the interaction of mosquito lifespan, EIP and vector competence is recognized, but there has, until now, not existed a method to evaluate this interaction with more than rudimentary comparisons [1], [2], [4], [17], [33], [34].

Instead of evaluating vectorial capacity and vector competence at discrete time points, as traditional use of vector competence data allows, vector competence can be modeled as a function of the rate of change over time in days, giving a value we term “effective vector competence (EVC).” This value is bounded by the survivability of the mosquito population, given that it includes p^N^ as a crucial evaluative parameter. This EVC can then be put back into the vectorial capacity equation as a parameter, resulting in a vectorial capacity curve as a function of time. Utilizing the EVC and vectorial capacity curve rather than single day or even day-by-day vector competence values accounts for the importance of the mosquito lifespan. When taken in concert, EIP (now a range of days), vector competence, and daily survival rate captures a more accurate picture of the natural transmission potential of a pathogen. Accordingly, we investigated the rate of change in vector competence, over a given interval, of dengue virus in *Aedes aegypti* mosquitoes, and the resulting EVC and vectorial capacity values, which resulted in a curvilinear function. The area under these curves represents the average vector competence bounded by mosquito lifespan and the average cumulative transmission potential of the arboviruses within a population of mosquitoes for a given time interval, respectively. Four strains of dengue virus were evaluated in this manner to demonstrate the value of this model based on our experimental data. Further we use previously published data on both West Nile virus and chikungunya virus to prove the validity our model.

The use of vectorial capacity to statistically compare virus transmission differences has been limited due to the inability to efficiently test differences, and often the statistical comparisons are limited to the vector competence data [2], [35], [36], [37]. Because vector competence is a parameter of proportion, each estimate of vectorial capacity contains within it an entire experiment aimed at estimating vector competence. Statistical methods that are readily available to researchers would require several replications of vectorial capacity estimates or artificial computer simulations. We used the calculated area under the curve for each virus strain in this study and calculated a corresponding variance estimate based on the inherent variance in the vector competence functions. We then used these estimates to test for differences in cumulative transmission potentials between strains of dengue virus based on our dynamic model.

The purpose of this modeling effort is to demonstrate that day-by-day comparisons of vector competence alone are not sufficient to offer consistent estimates of viral fitness. Additionally, the inclusion of the daily survival rate of the mosquito population makes the vector competence function much more relevant, as these bounds on EIP are important for fitness evaluation. Implicit in our calculation of a cumulative measure is the assertion that transmissibility at earlier time points is critical to characterizing epidemiologically relevant differences in viral strains, and that those differences are less apparent or lost when vectorial capacity is calculated with single day measures or even maximum measurements of vector competence at later times. Finally, because the single day value of EIP in the field is impossible to pinpoint and unlikely to be meaningful (outside of point source introduction of virus), a range of time covering the transmission critical period will more accurately represent what is happening under natural conditions. In order to more accurately evaluate viral fitness, we offer a model of cumulative vectorial capacity and effective vector competence to show that 1) single day comparisons are inadequate and 2) even with the collection of day-by-day vector competence values, a cumulative evaluation is needed.

## Materials and Methods

*Aedes aegypti* (Linnaeus) Rockefeller strain mosquitoes from the colony at Louisiana State University School of Veterinary Medicine were used in this experiment. Cartons containing approximately 100 individuals were kept in an environmental chamber at 28°C, 75–80% humidity, and subjected to a 16∶8 light:dark regime. Mosquitoes were provided water after emergence until the time of blood-feeding; no mortality due to sugar starvation was observed. After blood-feeding, mosquitoes were supplied with fresh water and 10% sucrose solution for the remainder of the experiment.

Four strains of dengue from Southeast Asia were utilized in this experiment to demonstrate the hypothesized behavior of a multiple strain system. Three strains of serotype 2 (16803, 1232 and 16681) and one strain of serotype 4 (LN 634441) were propagated by inoculating a T-75 flask of confluent Vero cells with 100 µl of viral stock for 15 minutes [23]. Ten mL of M199E medium with 10% newborn calf serum and 2% penicillin/streptomycin/fungizone was added. Flasks were incubated at 35°C with 5% CO_2_ for 6–8 days when they were harvested for virus at peak levels. Viral standard curves and concentrations were obtained via plaque assay as described previously before the beginning of the experiment and titers were verified throughout the experiment, including testing of blood meal titers, by qRT-PCR as previously described [38]. We used the SuperScript III One-step qRT-PCR kit (Invitrogen, Carlsbad, CA) as per manufacturer's instructions.

Mosquitoes were offered an infectious blood meal 3–5 days post emergence with an infectious titer of 10^6^ pfu/ml for all strains. The blood meal consisted of bovine blood in Alsevier's anticoagulant (Hemostat, Dixon, CA) mixed 2∶1 with a virus solution in a total volume of approximately 3 ml per carton, heated to 37°C and kept warm via the Hemotek device (Discovery Workshops, Arrington, Lanceshire, UK). Mosquitoes were allowed to feed for 45 minutes before the blood meal was removed. Mosquitoes were then sorted and only fully engorged females were kept; all others were discarded. Engorged females were identified by the presence of red blood in the abdomen, visible with the naked eye and these mosquitoes were our exposed cohort. Mosquitoes were then sampled at days 5, 7, and 9 post exposure to test for dissemination status. Sample sizes at each day are given in Table 1.

**Table 1:** Dissemination rates and samples sizes for 4 strains of dengue at 5, 7, and 9 days post exposure.

			Dissemination Rates (n)		
Serotype	Strain	Origin	5 dpe	7dpe	9dpe
2	1232	Indonesia, human	0 (19)	0.35 (17)	0.44 (18)
2	16803	Thailand, human	0 (18)	0.06 (15)	0.58 (12)
2	16681	Thailand, human	0.045 (22)	0.28 (25)	0.35 (31)
4	LN 634441	Malaysia, human	0 (11)	0 (11)	0.18 (11)

A disseminated infection where virus is present in legs and tissues other than the midgut, has been used to assess vector competence of dengue in *Aedes* mosquitoes, as well as other arboviruses in mosquito vectors. [14], [18], [39], [40]. Mosquito legs were removed and analyzed separately for infection from the bodies. Legs and bodies were put into separate vials containing 900 µl of BA-1 diluent [41] and then homogenized at 20 Hz for 2 minutes using a Tissuelyzer (Qiagen). RNA was extracted using the MagMax-96 kit (Ambion) on a King Fisher® nucleic acid extraction instrument according to the manufacturer's instructions (Thermo Scientific). After extraction, the samples were tested for the presence of dengue viral RNA via qRT-PCR using the following protocol: RT step (1 cycle) 48°C for 2 minutes, 95°C for 2 minutes; amplification and data recording step (40 cycles) 95°C 15 seconds, 60°C 30 seconds. Primers were designed and obtained via Integrated DNA Technologies (Table 2) with 5′ FAM fluorophore and 3′ Black-Hole quencher for DEN-2 and 5′ TAMRA fluorophore and 3′ Iowa-Black quencher for DEN-4. These primers and probes do not cross react, and are specific to only the intended serotype of dengue. Vector competence was calculated as the number of disseminated infections divided by the total number of mosquitoes exposed, as described above. All analyses and modeling was performed in SAS 9.13 (Cary, NC).

**Table 2:** Primer and probe sequences for dengue serotypes 2 and 4. All sequences are 5′ → 3′.

Serotype	Forward Primer Sequence	Reverse Primer Sequence	Probe Sequence
DEN-2	CAGGTTATGGCACTGTCACGAT	CCATCTGCAGCAACACCATCTC	CTCTCCGAGAACAGGCCTCGACTTCA
DEN-4	TTGTCCTAATGATGCTGGTCG	TCCACCTGAGACTCCTTCCA	TTCCTACTCCTACGCATCGCATTCCG

### Effective Vector Competence (EVC) and Cumulative Vectorial Capacity (cVC) Model

**Figure 1:**
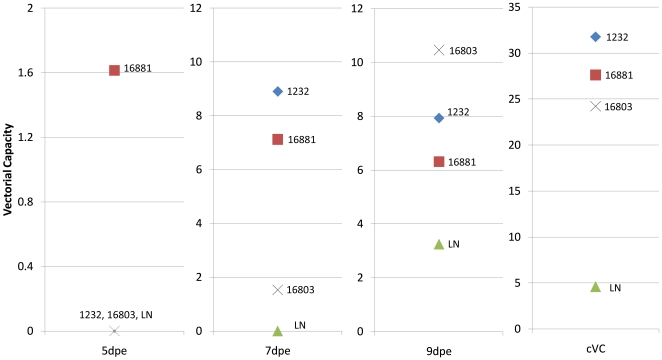
Vectorial capacity values for four strains of dengue at 5, 7, and 9 days post exposure as well as the cumulative vectorial capacity (cVC) values. The virus strain with the highest calculated fitness (i.e. transmissibility - VC) varies at each discrete time point, highlighting the difficulty of choosing a valid single EIP value. The cVC values take into account days (5, 7, and 9) and the daily survival rate of the mosquito (0.91), offering a more accurate measure of viral fitness.

Because we are interested in comparing viral characteristics only, we will hold **m**, as well as **a** (man biting rate) and **p** (probability of daily survival), constant. The values used in this effort are shown in Table 3. The assumptions of our model are a naïve end-host population and no significant vertical transmission of the pathogen within the vector. While vertical transmission has been observed with dengue in *Aedes ageypti*, levels are very low and an impact on transmission has not been definitively proven [42], [43]. Vector competence values used in this dengue vectorial capacity modeling effort are given in Table 3. Figure 1 shows traditional, single time point values of vectorial capacity calculated as in Eq. 1 and the comparison of our formulated cumulative vectorial capacity (cVC) described below. This figure highlights the importance of accounting for variation of vector competence over the course of time, as the rank of fitness as judged by vectorial capacity swaps from day to day.

**Table 3:** Parameters of the Vectorial Capacity Equation held constant.

Parameter	Value	Reference
Mosquito Density (m)	1.9	[48]
Man Biting Rate (a)	3.125	[49]
Probability of Daily Survival (p)*	0.91	[50]

Parameter values of *Aedes aegypti* obtained from literature and held constant when calculating the Vectorial Capacity over a series of days, N, and corresponding vector competence values, b_N_.

*Denotes average of presented data.

We devised a method that uses the rate of change in vector competence as part of the vectorial capacity equation. For each set of dissemination rates over a given interval meant to represent the EIP, a relationship defining b as a function of this interval was devised: 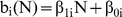
(2)where b_i_(N) is the function for strain i; β_1_ is the determined change in b per unit change in N (slope of the line); N is day post exposure, and β_0_ is the y-intercept. This line represents the rate of change in dissemination rates over time and the variance inherent to this line will be used to construct variance estimates. The interval over which this line is constructed has a lower limit of time *a* and an upper limit of *z*. Using the rate of change function for each strain *i*, EVC is defined as:
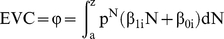
(3)


And cVC is now defined as:
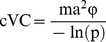
(4)


**Figure 2:**
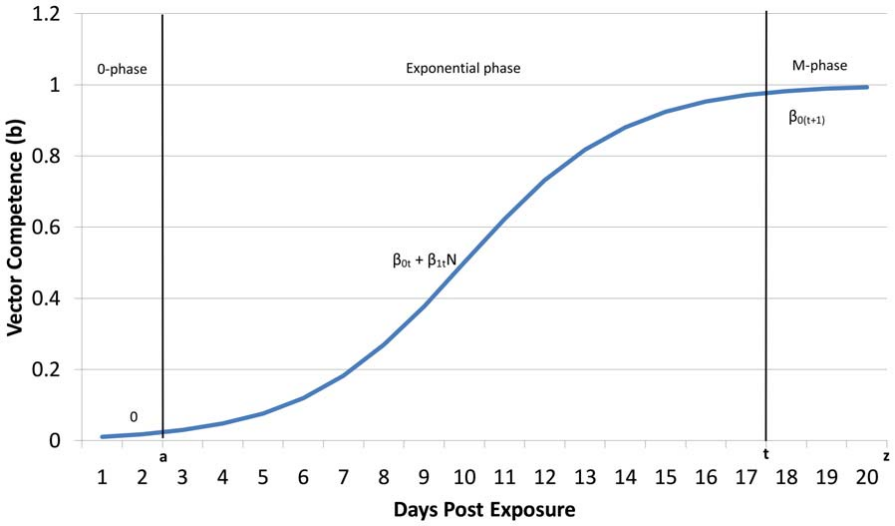
Vector competence dynamics. The complete lack of dissemination during the 0-phase will contribute 0 to the cumulative vectorial capacity calculation, while the functions in both the exponential growth and M- phases will be incorporated into the calculation of cVC. During the exponential growth phase, the linear function of vector competence growth will be incorporated in the cVC function, while in the M-phase, where dissemination rates have reached a plateau, only the y-intercept based on the value of the dissemination rate plateau value is necessary.

As vector competence is a proportion, it is asymptotic at 0 and at 1 and the function of change is a sigmoidal (S) curve often analyzed via logistic regression. A sigmoidal curve is characterized by a plateau before (minimum  = 0) and after (maximum = M) a phase of exponential growth within the interval [a,t]sf where a is the beginning of the exponential growth phase and t is the end of the exponential phase(Figure 2). The interval (t,z] is the M-phase. At both the 0- and M-plateau phase of this curve, the rate of change of vector competence is either negligible or zero. During the 0-plateau phase, there is no mathematical contribution to the calculation of cVC, but during the M-plateau, the changes in cVC will largely be driven not by vector competence, but by the survival rate of the vector. If the interval of experimentation does not include the M-plateau phase, then vectorial capacity is calculated as above, using the linear function b_i_(N). If the M-phase is sufficient enough to contribute, then cVC is calculated by adding the areas under the curve of the exponential and M-phases (see Supporting Information S1 for details).

As the dengue strains used in this study did not reach M-phase over the experimental time interval (days 5–9 post exposure), the cVC was calculated from the exponential phase only, as in Supporting Information S1. Corresponding variance estimates are calculated using the variation inherent in the linear function, b*_i_*(N), from the exponential growth phase of vector competence. One of the benefits of using linear regression equations to define cVC rather than directly incorporating the logistic function is the calculation of an accurate variance estimate, whereas logistic regression functions are based on maximum likelihood and thus variances often do not converge on true values of variance and confidence levels are approximations. Variance calculations are given in Supporting Information S1. The use of areas under the curve and corresponding variance estimates to test for differences has been established [44], but use in vector-borne disease transmission comparisons has not been explored. The results of this method for four dengue strains are given in Table 4.

**Table 4:** Results from the cumulative vectorial capacity method for 4 strains of dengue.

Strain	Lower 95% Confidence Limit of Difference*	cVC	Std. Err. (cVC)	Upper 95% Confidence Limit of Difference*
1232	n/a	31.7616	6.66	n/a
16803	−24.3807	24.2305	5.92	12.4718
16681	−21.7204	27.6219	5.54	12.8051
LN 634441	−42.1138	4.69539	3.00	−12.5657

Cumulative vectorial capacity (cVC) estimates obtained from our model of integration and the associated variance estimates for each strain.

*Indicates the 95% lower and upper confidence limits for the difference between each strain and D2 1232.

### Statistical Test of Differences

We also provide a method for statistically testing the cVC. It is important to note that cVC itself is not a mean or a sum, but a fine scale sum of means. To demonstrate, we show here a simple sum, though in actuality we integrate over the interval to capture the continuous rates of change of vector competence. Let E(VC_x_) be the mean vectorial capacity at day X, then:
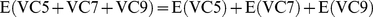
(5)


The importance of considering the cumulative transmission potential of a mosquito population is depicted in Figure 1 where the strain of DENV with the highest dissemination rate at N = 9 (D2 16803) has the third highest cVC.

To test for differences between strains, confidence intervals about the mean differences in cVC estimates should be constructed based on an acceptable confidence level and the appropriate degrees of freedom (based on the sample size of the vector competence function). If the data are sufficiently normal, using critical values from the Student's t-distribution is acceptable. However, a robust alternative if data are not sufficiently normal is the construction of confidence intervals based on a t-like distribution of the differences. Area estimates and variances are obtained as above after bootstrap re-sampling with replacement for 1000 bootstrap iterations. The differences between these simulated area estimates and variances per strain are then used to develop a t-like distribution which, given the number of bootstraps, is normally distributed. A t-like distribution has the properties of the Student's t-distribution, but the mean is shifted from 0 to a value dependent on the comparisons made. The distribution of the differences is constructed and the values of the 2.5 and 97.5 percentiles of this t-like distribution (t_.025_ and t_.975_, respectively) are obtained and used to construct a 95% confidence interval of the difference. For example, to compare strains 1 and 2, using the data obtained experimentally and the t-like distribution from the bootstrap efforts, the upper and lower confidence limits are obtained by:
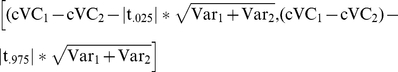
(6)


The values cVC_1_, cVC_2_, Var_1_, and Var_2_ are the values obtained from the original computations of cVC based on the experimental data and b*_i_*(N); the bootstrapping is to facilitate the t-like distribution and produce the values of t_.025_ and t_.975_ only. As in all interval tests of hypotheses, if the null value (i.e. a difference of 0) is contained within this interval, no significant difference exists between the compared strains. Results of pair wise testing for strains of dengue are given in Table 4.

By calculating the rate of change of vector competence and pairing this with the other parameters of the vectorial capacity equation, especially the survival function, a more accurate understanding of the comprehensive differences in the potential for transmission of arboviruses is obtained. For the sake of brevity, we present only the tested differences in strains with respect to an arbitrarily designated reference strain, D2 1232. We constructed 95% confidence intervals using cVC estimates and standard error estimates to test for significant differences between the reference strain and the other four strains. Only strain LN 634441 was significantly different from the reference strain (Table 4). This demonstrates the ability of our model to distinguish between significant and non-significant differences. Using the definition of vectorial capacity and assuming a system of perfect transmission where every disseminated infection results in a successful transmission event, for every 10 cases of DENV transmitted from this population of mosquitoes that is attributable to strain 1232, one would expect only ≈2 to arise from strain LN 634441.

### Characterization of Fitness in the Vector

To further characterize differences between dengue strains in the context of viral fitness, we devised a displacement index (DI). Viral fitness is a measure of the relative replicative abilities of a viral strain to a reference strain [45]. Vector competence can be used as a measure of relative fitness, and thus so can vectorial capacity and cVC [2]. To isolate viral characteristics, and using the properties of integration, the “entomological” parameters can be moved to the outside of the integral.

This displacement index is then defined as:
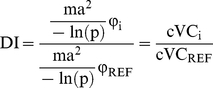
(7)


When two strains (*i* and a reference strain *REF*) are co-circulating in a single population of mosquitoes which is assumed to be homogeneous, the parameters considered to be intrinsic to the vector effectively cancel out. The DI is thus interpreted as a measure of the capability of a newly introduced strain to displace the established, dominant circulating strain:
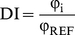
(8)


When the entomological parameters cancel out, the viral differences, measured as effective vector competence, are what determine whether one strain is capable of displacing the dominant circulating strain or serotype: the capability and speed of dissemination. Our model of EVC and cVC captures these parameters in a more thorough measurement, and as the DI utilizes cVC and/or EVC, it is likewise a more complete measure of viral fitness with regards to transmission. If entomological and vertebrate parameters of the vectorial capacity equation are held constant, when DI >1, there exists some intrinsic characteristic of that viral strain that will infer on the displacing strain a competitive advantage. In this way, the DI can be used to compare intrinsic viral fitness of viral strains within a vector population or can be used to determine the potential of a new virus to invade and hijack a vector population where an established arbovirus has sustained transmission.

## Results and Discussion

Expectation has been that arbovirus strains with lower vector competence at late EIPs will be at a competitive disadvantage and an arbovirus strain with the highest ultimate value of **b** should out-compete strains with lower dissemination rates at some fixed EIP. For example, the strain of West Nile virus originally introduced to North America (NY99) has since been displaced by another strain (WN02) which has a shorter extrinsic incubation period within its primary vectors, the *Culex* spp. mosquitoes [13]. Alone, the values of vector competence and EIPs are informative, however we detected highly variable times to initial and maximum dissemination and dissemination rates based on the strain of dengue virus. Taken at each time point, strain differences can be seen, but no clear pattern emerges for definitive conclusions. For example, if we were to compare the fastest start of dissemination, D2 16681 is the only strain with any dissemination at day 5. At day 7, however, this strain is out disseminated by D2 1232 which then falls behind D2 16803 at day 9 post exposure. The highest ultimate dissemination is seen in D2 16803 (58% at 9 dpe). These differences across time points show the difficulty in assessing fitness at discrete points. Further, though D2 16803 ultimately achieves the highest dissemination rate, because of the force of the survival function on vectorial capacity, this strain does not possess the highest cVC and therefore is not necessarily the most efficiently transmitted strain. In fact, D2 16803 did not have cVC above D2 1232 or D2 16681 which only had dissemination rates of 44% and 35% at day 9, respectively. D4 LN 634441 lagged at all time points and was the only strain to be accurately assessed at each single time point, though this is attributable to the overall inefficiency of the strain.

While we use testing of legs to extrapolate transmission rates, we recognize that there has been no definitive evidence that proves this measure does not overestimate vector competence. There is no evidence to support the supposition that this overestimation would be differential across strains. The lack of an accessible transmission model for dengue has confounded such investigations, and this further highlights the importance of moving towards such a model.

As vector competence is a dynamic function of time, selection of an appropriate EIP (or range thereof) for testing is critical [46]. However, it is also important to note that at later time points, survivorship of the mosquito cohort declines. The effect of the interaction of vector competence and declining survivorship has on estimates of vectorial capacity had not been rigorously explored. A strain that results in a smaller proportion of disseminated infections, but that invades much faster will infiltrate the mosquito population and perhaps render a portion of the vertebrate population immunologically unavailable to the strains with slower kinetics, as well as take advantage of a higher proportion of surviving vectors, a relationship not accurately reflected by simple vector competence comparisons. This demonstrates that there is a trade-off in vector competence and EIP, which this model accurately captures. This model should retain its accuracy and usefulness when comparing across vector populations and/or species by varying the other parameters according to the vector(s) of interest.

The argument that vectorial capacity values are most informative when used in a comparative way is not new [6]. A decisive, interpretable method for doing such a comparisons has until now, been unavailable. With this data, we demonstrate how the varying values of vector competence can be used to calculate the true magnitude of transmission potential and that these cumulative values are the basis of accurate tests of differences in these potentials.

**Figure 3:**
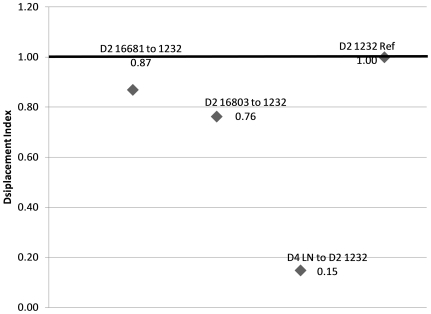
The displacement index of three dengue strains (D4 LN 63441, D2 16681, D2 16803) compared to the reference strain (D2 1232) shows that no displacement should occur if these strains were to co-circulate.

In complex vector-borne disease transmission systems such as dengue, where multiple serotypes of an arbovirus co-circulate, understanding the relative kinetics of transmission of co-circulating strains and serotypes is a vital part of understanding the overall transmission. This is especially so in dengue endemic areas where serotype switching events have been linked to more severe disease outbreaks [47]. With the understanding that entomological parameters cancel out in our calculation of the DI, we formulated a comparison of relative fitness. In a theoretical system where D2 1232 is established as the dominant strain, and the other four dengue virus strains have been introduced and now co-circulate, none of the strains are capable of overtaking the system and displacing strain 1232. Conversely, if LN were the reference and 1232 the invader, 1232 would have the potential to displace LN with a ratio of infectious bites of 10∶4. Excess secondary vertebrate cases of strain 1232 translate into enhanced transmission potential to naïve vectors, continuing the transmission cycle with more force than the other strains and thus perpetuating its dominance (Figure 3).

To further validate our model, we used data from Moudy et al. that investigated the differences in vector competence between the NY99 and WN02 strains of West Nile virus [13]. The displacement index of WN02 in relation to NY99 is 2.14, a value that is supported by the invasion of WN02 into the West Nile transmission system and its complete displacement of the established NY99 strain. A pathogen such as WNV which has multiple vector species, can add a new level of complexity to the model. In such cases, the entomological parameters would have a great impact on the transmission system. But like the cVC model in general, the DI can be used to make comparisons made across different populations or species of vectors.

Similarly, we demonstrate the use of the displacement index using data from two strains of chikungunya virus isolated from La Reunion Island during the 2005-2006 epidemic [16]. The mutation in the envelope changed an alanine to a valine and increased the efficiency of the virus within the vector *Aedes albopictus* [16]. The displacement index of the viral strains (E226V:E226A) in *Ae. albopictus* was calculated to be 1.91, and further demonstrates the use of the displacement index as a measure of both viral fitness within a vector and a means of comparing transmission potential.

As these data indicate, our cVC methodology gives a more accurate measure of the magnitude of transmission potential, owing to the fact that it collapses several informative parameters into a single, standardized measure. In addition, it allows for direct statistical tests of differences in cumulative vectorial capacity where there has previously been none. The DI provides a scaled index by which viral fitness can be measured and compared, an assessment which further characterized the four dengue strains used. In addition, as the historical events highlight, the DI could indicate the potential emergence of new pathogen threats to public health, economy, and national security. The validation using West Nile and chikungunya data gives us confidence that this method will be a useful epidemiologic measure and future directions include investigations of field isolated dengue from endemic areas.

In summary, the cVC model along with the DI provides a conceptual and methodological basis by which virus fitness differences can be evaluated within an epidemiologically satisfying framework. This methodology will be additionally useful in retrospective characterizations of observed viral genetic and phenotypic differences detected during past epidemics, where attribution of the emergence event is of interest.

## Supporting Information

Supporting Information S1 (DOC)
