# Supplementary material for: Estimating the Magnitude and Direction of Altered Arbovirus Transmission Due to Viral Phenotype
Source: PLoS One. 2011 Jan 27;6(1):e16298. doi: 10.1371/journal.pone.0016298 (PMC3029343; doi:10.1371/journal.pone.0016298)
Supplement: Supporting Information S1 — (DOC) [file pone.0016298.s001.doc]

Supplement

For strain i, the cumulative vectorial capacity (cVC) is the sum of the components of the exponential and M-phases:

Exponential Growth Phase:

Where
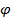
 is the effective vector competence (EVC) parameter.

$$Area_{Exponential-Phase}=\frac{ma^{2}}{-\ln(p)}[\beta_{1i}Z_{0}+\beta_{0i}Z_{1}]$$

Definition S1

M-phase:

$$Area_{M-Phase}=\frac{ma2}{-\ln(p)}\int_{t}^{z} p^{N}(\beta_{0i})dN=\frac{ma2}{-\ln(p)}[\beta_{01}\int_{t}^{z} p^{N}dN]$$

Definition S2

cVC then, is given by:

$$cVC=Area_{Exponential-Phase}+Area_{M-phase}$$

Eq. S1

To calculate variance estimates:

Let: $\widehat{\text{β}}i=\left(\begin{matrix} \beta_{0i} \\ \beta_{1i} \end{matrix}\right)=(\text{X`X})^{-1}\text{X`}\widehat{\text{b}}$

Definition S4

Where $\text{X}=\left[\begin{matrix} 1 \\ 1 \\ 1 \end{matrix}\begin{matrix} \\ \\ \end{matrix}\begin{matrix} N_{1} \\ N_{2} \\ N_{i} \end{matrix}\right]$ where N_i_ is the time value at sampled time point t

Definition S5

And $\widehat{\text{b}}=\left[\begin{matrix} b_{1} \\ b_{2} \\ b_{n} \end{matrix}\right]$ where b is the observed proportion of disseminated infections at time t

Definition S6

Then for strain i at time points 1 to t:

$$Var(\widehat{\text{β}_{\text{i}}})=\text{(X`X)}^{\text{-1}}\text{X`}\left[diag\left(\frac{b_{ti}(1-b_{t1})}{n_{t1}},\cdots,\frac{b_{tn}(1-b_{tn})}{n_{tn}}\right)\right]\text{X(X`X)}^{\text{-1}}$$

Definition S7

And the variance of the area estimate for strain *i* is:

$$Var(\widehat{Area_{i}})=\left(\frac{ma^{2}}{-\ln(p)}\right)^{2}(Z_{0},Z_{1})Var(\widehat{\text{β}_{\text{i}}})\left[\begin{matrix} Z_{0} \\ Z_{1} \end{matrix}\right]$$

Eq. S2
